# Retrospective Analysis of Cholera/Acute Watery Diarrhea Outbreaks in Ethiopia From 2001 To 2023: Incidence, Case Fatality Rate, and Seasonal and Multiyear Epidemic Patterns

**DOI:** 10.1093/cid/ciae236

**Published:** 2024-07-12

**Authors:** Yeshambel Worku Demlie, Abel Gedefaw, Yeonji Jeon, Dejene Hailu, Tomas Getahun, Ondari D Mogeni, David Mukasa, Geun Hyeog Jang, Gi Deok Pak, Deok Ryun Kim, Edlawit Mesfin Getachew, Biruk Yeshitela, Samuyel Ayele Abebe, Moti Edosa, Mesfin Wossen, Mekonnen Teferi, Se Eun Park

**Affiliations:** Diseases Surveillance and Response Directorate, Ethiopia Public Health Institute, Addis Ababa, Ethiopia; Clinical, Assessment, Regulatory, Evaluation (CARE) Unit, International Vaccine Institute, Seoul, Republic of Korea; College of Medicine and Health Sciences, Hawassa University, Hawassa, Ethiopia; Clinical, Assessment, Regulatory, Evaluation (CARE) Unit, International Vaccine Institute, Seoul, Republic of Korea; Clinical, Assessment, Regulatory, Evaluation (CARE) Unit, International Vaccine Institute, Seoul, Republic of Korea; School of Public Health, Hawassa University, Hawassa, Ethiopia; Clinical Trials Directorate, Armauer Hansen Research Institute, Addis Ababa, Ethiopia; Clinical, Assessment, Regulatory, Evaluation (CARE) Unit, International Vaccine Institute, Seoul, Republic of Korea; Biostatistics and Data Management Department (BDM), International Vaccine Institute, Seoul, Republic of Korea; Biostatistics and Data Management Department (BDM), International Vaccine Institute, Seoul, Republic of Korea; Biostatistics and Data Management Department (BDM), International Vaccine Institute, Seoul, Republic of Korea; Biostatistics and Data Management Department (BDM), International Vaccine Institute, Seoul, Republic of Korea; Clinical Trials Directorate, Armauer Hansen Research Institute, Addis Ababa, Ethiopia; Bacterial and Viral Disease Research Directorate, Armauer Hansen Research Institute, Addis Ababa, Ethiopia; Data Science Division, Armauer Hansen Research Institute, Addis Ababa, Ethiopia; Diseases Surveillance and Response Directorate, Ethiopia Public Health Institute, Addis Ababa, Ethiopia; Diseases Surveillance and Response Directorate, Ethiopia Public Health Institute, Addis Ababa, Ethiopia; Clinical Trials Directorate, Armauer Hansen Research Institute, Addis Ababa, Ethiopia; Clinical, Assessment, Regulatory, Evaluation (CARE) Unit, International Vaccine Institute, Seoul, Republic of Korea; Department of Global Health and Disease Control, Yonsei University Graduate School of Public Health, Seoul, Republic of Korea

**Keywords:** cholera, incidence, case fatality rate, seasonality, Ethiopia

## Abstract

**Background:**

The Ethiopian government has developed the multisectoral cholera elimination plan (NCP) with an aim of reducing cholera incidence and case fatality rate (CFR). To better understand and monitor the progress of this plan, a comprehensive review of national cholera epidemiology is needed.

**Methods:**

Reported data on cholera/acute watery diarrhea (AWD) cases in the past 20 years were extracted from the Ethiopian Public Health Institute and World Health Organization databases. Descriptive statistics, Pearson χ^2^, and logistic regression analyses were conducted.

**Results:**

From January 2001 to November 2023, a total of 215 205 cholera/AWD cases, 2355 deaths with a cumulative CFR of 1.10% (95% confidence interval [CI], 1.092–1.095), and a mean annual incidence rate of 8.9/100 000 (95% CI, 6.5–11.3) were reported. Two major upsurges of cholera epidemics were found in the last two decades with mean attack rate (AR) of 20.57/100 000 in 2006–2010 and 14.83/100 000 in 2016–2020. Another resurgence of outbreaks occured in 2021–2023 (mean AR, 8.63/100 000). In 2015–2023, 54.0% (53 990/99 945) of cases were aged 15–44 years. National cholera CFR (3.13% [95% CI: 2.1–4.5]) was the highest in 2022. The 2015–2023 cumulative cholera CFR was different across regions: Benishangul Gumuz (6.07%), Gambela (1.89%), Sidama (1.42%), Southern Nation, Nationalities, and Peoples’ (1.34%), Oromia (1.10%), and Amhara (1.09%). Cholera/AWD patients in older adults (≥45 years), severe dehydration, peak rainy season (June–August), and outpatients were associated with higher risk of death.

**Conclusions:**

Cholera has been a public health problem in Ethiopia with case fatalities still above the global target. Case management needs to be improved particularly in outpatients and older populations. Outbreak preparedness should be rolled out well in advance of the typical rainy seasons. Significant investments are essential to advance the cholera surveillance system at healthcare setting and community level. Underlying factors of cholera deaths per areas should be further investigated to guide appropriate interventions to meet the NCP target by 2028.

## INTRODUCTION

Cholera remains an important public health problem worldwide, especially in countries with limited water, sanitation, and hygiene (WaSH) and inadequate health system to detect and treat patients in a timely manner and control transmissions. The global burden of cholera remains high with an estimated 1.3 billion people at risk and an annual incidence of 2.9 million (uncertainty range: 1.3–4.0 million) and 95 000 deaths (uncertainty range: 21 000–143 000), with sub-Saharan Africa and Southern Asia accounting for 60% and 29% of the burden, respectively [1, 2]. The true global burden of cholera is unknown due to possible under-reporting [3]. Ethiopia is one of the countries that has experienced endemic and epidemic cholera for decades [4–7]. The government of Ethiopia has published the Guideline on Cholera Outbreak Management in 2011 [8] and the National Guildeline for Cholera Surveillance and Outbreak Response in 2022 [9]. Since 2019, the oral cholera vaccine (OCV) has been used widely by the local government in response to a series of cholera outbreaks across multiple regions in Ethiopia [10]. A comprehensive multisectoral national cholera plan (NCP) has been also developed and made available since 2022 [11]. Despite these government efforts, multiple cholera outbreaks have been persistently affecting local populations. However, there is lack of understanding on the trends of cholera epidemiology in Ethiopia, including national/regional outbreak patterns, incidence, case fatalities, seasonality, and underlying risk factors related to cholera deaths. It is important to identify gaps and challenges in cholera surveillance and case management in order to institute appropriate and targeted interventions. Here, we aimed to present a comprehensive overview of cholera epidemiology in the entire country during the past two decades.

## METHODS

### Study Setting

Ethiopia is a landlocked country in the Horn of Africa located at 9.1450°N, 40.4897°E. It shares borders with Eritrea to the north, Djibouti to the northeast, Somalia to the east and northeast, Kenya to the south, South Sudan to the west, and Sudan to the northwest. Ethiopia has a total area of 1 128 571 km^2^ with the population density reaching 107 people per km^2^ in 2021 [12]. The population size of the entire country reached over 123 million in 2022 [12]. At the national level, the use of a basic water source increased from 18% in 2000 to 50% in 2016, and open defecation declined from 82% to 32% over the same period. However, in 2016, only 6% of households had access to a basic sanitation facility, and 40% of households had no handwashing facilities [13]. As of 2024, the country has 12 regions and 2 city administrations.

### Cholera Surveillance, Outbreak Investigation, and Reporting in Ethiopia

Cholera is a mandatory notifiable disease in Ethiopia. Continuous healthcare facility-based and community-level surveillance are implemented using the Integrated Disease Surveillance and Response system. The case detection and reporting system is according to the national guidelines developed, first in 2011 [8] and updated in 2022 [9]. Prior to 2011, there was no official government document on cholera case definition or cholera surveillance system at the national or regional level. A suspected case of cholera is defined as any person ≥5 years of age with profuse acute watery diarrhea (AWD) and vomiting. A confirmed case of cholera is defined as a suspected case with *Vibrio cholerae* O1 or O139 isolated from stool, a sufficient threshold for an outbreak to be declared. Cholera outbreak declarations are made in cholera-epidemic areas, when a patient aged ≥5 years who develops AWD, with or without vomiting, is detected; and in an area where cholera is not known to be prevalent, when a patient aged ≥5 years who develops severe dehydration or dies from AWD is detected [8]. When a suspected cholera case is reported, a multidisciplinary outbreak investigation team—rapid response team—is organized at the Ethiopian Public Health Institute (EPHI) or district level, and an outbreak investigation initiated within 3 hours [8, 9].

The recently revised National Guideline for Cholera Surveillance and Outbreak Response published in 2022 [9] incorporates significant changes compared to the previous 2011 guideline [8]. The key additions to this guideline include the following: (1) OCV is integrated as a crucial component of cholera control interventions; (2) the age cut-off limit for case definition criteria has been reduced to 2 years from the previous threshold of 5 years; a patient aged ≥5 years develops severe dehydration or dies from AWD (2011 guideline), changed to ≥2 years with profuse AWD and vomiting (2022 guideline); (3) specific guidance is provided for managing cholera outbreaks within internally displaced populations, recognizing the unique challenges in such settings; and (4) the guideline underscores the importance of establishing a robust cross-border surveillance system, emphasizing the need for collaborative efforts to monitor and manage cholera outbreaks across borders [9]. The term ‘AWD’ instead of cholera was used before 2019 [14]. The determination of the case fatality rate (CFR) relies on data gathered from both facility- and community-based surveillance systems [8, 9]. However, the reported CFR may closely align with the fatality rate derived from facility-based data, as there is a notable risk of underreporting associated with community-based deaths.

### Study Design, Data Source, and Period

A comprehensive retrospective analysis of the national cholera/AWD outbreaks in Ethiopia in the recent 2 decades was conducted. National data on cholera/AWD from January 2001 until November 2023 was collected from the EPHI government and WHO databases, and data curation and analysis were conducted. We used the WHO cholera database [15] for the 2001–2014 incidence and CFR estimations, due to the lack of comprehensive documentation of cholera line-listing data at the national level before 2015. For calculation of incidence rates, we used annual population estimates projected by the Ethiopian Central Statistics Agency based on the 2007 national census data [16]. Detailed descriptive analysis at the national and regional levels was conducted using the EPHI national cholera line-listing from January 2015 to November 2023. This dataset included basic epidemiological information such as date of cholera outbreak, age, sex, district, clinical outcome (death), level of dehydration, and diagnostic modality.

### Data Analysis

Descriptive analysis was conducted to summarize the epidemiological characteristics. The annual cholera incidence rate was estimated using the total number of cholera/AWD cases per 100 000 population. The annual CFR was calculated based on the cholera deaths out of total cholera cases reported. A 5-year mean annual cholera incidence and CFR were also estimated. Bivariate and multivariate logistic regression was conducted to evaluate variables associated with CFR. Statistical analysis was performed using SPSS version 26 software.

### Ethical Consideration

This study used the already available secondary aggregated public health surveillance data. Therefore, no institutional review board approval was necessary, sought, or obtained.

## RESULTS

### Descriptive Epidemiological Characteristics of Reported Cholera/AWD Cases From 2001 to 2023

In the last 2 decades, 215 205 cholera/AWD cases and 2355 deaths with a cumulative CFR of 1.10% (95% confidence interval [CI]: 1.092–1.095) were reported (Table 1). The highest case counts were reported during 2006, 2007, 2009, 2016, 2017, and 2023 with >20 000 AWD/cholera cases per year; followed by the outbreak in 2020 that recorded >15 000 cases. In the recent 5 years, the highest number of cases was reported in 2023 with nearly 30 000 cases.

**Table 1.** Annual Cholera Cases, Deaths, Incidence Rate, and Case Fatality Rate Reported in Ethiopia During 2001–2023

Year	Estimated Population^a^	AWD/Cholera Cases	Attack Rate/100 000	Deaths, No.	CFR,^c^ %
2001^b^	n.a	0	0	0	n.a
2002^b^	n.a	0	0	0	n.a
2003^b^	n.a	0	0	0	n.a
2004^b^	n.a	16	1.4	0	0.00
2005^b^	n.a	0	0	0	n.a
2006^b^	n.a	54 070	48.24	575	1.06
2007^b^	n.a	24 121	21.52	272	1.13
2008^b^	n.a	3862	3.5	23	0.60
2009^b^	n.a	31 509	28.11	434	1.38
2010^b^	n.a	1682	1.50	21	1.25
2011^b^	n.a	0	0	0	n.a
2012^b^	n.a	0	0	0	n.a
2013^b^	n.a	0	0	0	n.a
2014^b^	n.a	0	0	0	n.a
**2001–2014 total**	n.a	**115 260**	n.a	**1325**	**1.15**
2015	90 668 000	227	0.25	2	0.88
2016	92 931 000	29 871	32.14	186	0.62
2017	95 223 000	20 509	21.54	150	0.73
2018	97 540 000	3259	3.34	42	1.29
2019	99 880 000	2271	2.27	26	1.14
2020	102 235 000	15 167	14.84	219	1.44
2021	104 606 000	696	0.67	8	1.15
2022	106 983 000	862	0.81	27	3.13
2023	109 372 000	27 101	24.4	370	1.4
**2015–2023 total**	n.a	**99 945**	n.a	**1030**	**1.03**
**2001–2023 grand total**	n.a	**215 205**	n.a	**2.355**	**1.10**

Abbreviations: AWD, acute watery diarrhea; CFR, case fatality rate; n.a, not applicable. The bold values refer to the total or subtotal amount.

^a^Source of estimated population during 2015–2023 is the Ethiopian Central Statistics Agency.

^b^Data source: World Health Organization (WHO) database. The presented cases, attack rate, and CFR were extracted from the WHO data source; thus, not calculated by the authors of this manuscript.

^c^n.a in this column shows that CFR couldn't be calculated as there were no cholera cases.

### Descriptive Epidemiological Characteristics of the Recent 2015–2023 Cholera/AWD Cases

Between 1 January 2015 and 30 November 2023, a total of 99 945 cases and 1030 deaths were reported per the EPHI national cholera line-listing (Table 2). Of these 99 945 cases, 55 299 (55.3%) were male. In terms of the age-stratified cases reported during this period, 53 990 (54.0%) were aged between 15 and 44 years, 18 273 (18.3%) between 5 and 14 years, 16 215 (16.2%) aged ≥45 years, and 11 460 (11.5%) aged <5 years.

**Table 2.** Descriptive Epidemiological Characteristics of Cholera Cases in Ethiopia During 2015–2023

Characteristic	Category	Frequency, No.	Percentage, %
Sex (n = 99 945)	Female	44 646	44.7
	Male	55 299	55.3
Age (n = 99 938)	<5 y	11 460	11.5
	5–14 y	18 273	18.3
	15–44 y	53 990	54.0
	≥45 y	16 215	16.2
Region (n = 99 945)	Addis Ababa	7903	7.9
	Afar	6206	6.2
	Amhara	13 366	13.4
	Benishangul Gumuz	642	0.6
	Dire Dawa	873	0.9
	Gambela	211	0.2
	Harari	357	0.4
	Oromia	22 688	22.7
	Sidama	1833	1.8
	SNNPR	20 119	20.1
	Somali	19 010	19.0
	Tigray	6738	6.7
Reported period (n = 99 945)	2015	230	0.2
	2016	29 871	29.9
	2017	20 509	20.5
	2018	3259	3.8
	2019	2271	2.3
	2020	15 167	15.2
	2021^a^	696	0.7
	2022^a^	841	0.8
	2023	27 101	27.1
Outbreak reported season (n = 99 945)	March–May (Belg)^b^	21 015	21
	June–August (Kiremt)^c^	43 236	43.3
	September–November (Mehir)^d^	25 594	25.6
	December–February (Bega)^e^	10 100	10.1
Dehydration status at presentation (n = 99 945)	Not documented	2077	2.1
	No	13 662	13.7
	Some	36 340	36.4
	Severe	47 866	47.9
Sample was taken for laboratory diagnosis (n = 99 945)	Yes	8005	8.0
	No	86 096	86.1
	Unknown	5844	5.8
Diagnostic modality (n = 99 945)	RDT	7118	7.1
	Culture	851	0.9
	Both RDT and culture	52	0.1
	Clinically suspected case	86 080	86.1
Cholera test positivity out of samples taken (n = 8021)	RDT positive	6499	91.3
	Culture positive	624	73.3
	Both RDT and culture positive	100	100

Abbreviations: y, years; RDT, rapid diagnostic test; SNNPR, Southern Nation, Nationalities, and Peoples’ Region.

^a^The low number of cases reported in 2021–2022 may be due to the coronavirus disease 2019 (COVID-19) pandemic measures, such as social distancing, and the overstretched health system and surveillance capacity.

^b^Spring with a relatively rainy season in some parts of the country.

^c^Summer with a major rainy season all over the country.

^d^Spring with a relatively dry season.

^e^Autumn with a dry season.

**Figure 1.**
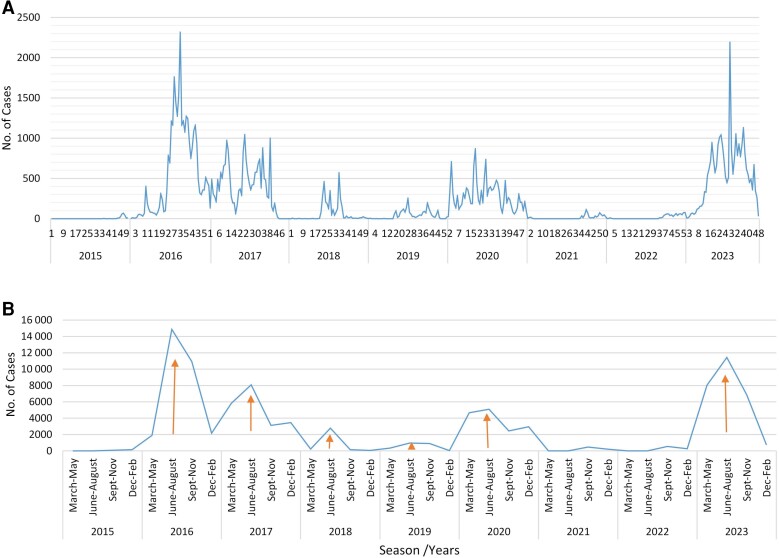
Cholera epidemic curves and seasonal patterns in Ethiopia. *A*, Successive cholera epidemic waves in Ethiopia every year from 2015 to 2023. The presented epi-weeks exhibit the magnitude and persistence of cholera outbreaks in the country. *B*, Seasonal pattern of cumulative cholera case counts reported by the major seasons in Ethiopia from 2015 to 2023.

During this period, cholera/AWD outbreaks in Ethiopia peaked, especially in 2016 (29 871/99 945 [29.9%]) and 2017 (20 509/99 945 [20.5%]) (Table 2 and Figure 1*A*). This was followed by the subsequent 2 years of lowered case numbers, though still thousands of annual cases were reported: 3259 (3.8% of 99 945) in 2018 and 2271 (2.3% of 99 945) in 2019. Then, cholera/AWD cases peaked significantly again in 2020, with thousands of additional annual cases (15 167/99 945 [15.2%]). The number of reported cholera/AWD cases declined in 2021 (696/99 945 [0.7%]) and 2022 (841/99 945 [0.8%]) during the COVID-19 pandemic but again peaked in 2023 (27 101/99 945 [27.1%]). In terms of seasonality (Figure 1*B*), cholera cases were reported throughout the year, but more in June–August (Kiremt; summer with a major rainy season all over the country) with 43.3% (43 236/99 945) of cases reported in this season during 2015–2023. Next, September–November (Mehir; spring with a relative dry season) and March–May (Belg; spring with a relative rainy season in some parts of the country) exhibited 25.6% (25 594/99 945) and 21.0% (21 015/99 945) of cases, respectively. December–February (Bega; autumn with a dry season) showed relatively lower number of cases (10 100/99 945 [10.1%]). The epidemiological curve (Figure 1*A*) exhibited persistent cholera outbreaks throughout the year (especially in 2016, 2017, 2020, and 2023) or outbreaks controlled and limited to few months (in 2018 and 2021). In those years where cholera persisted throughout the entire year, the weekly reported cases exceeded 500 (Figure 1*A*).

**Figure 2.**
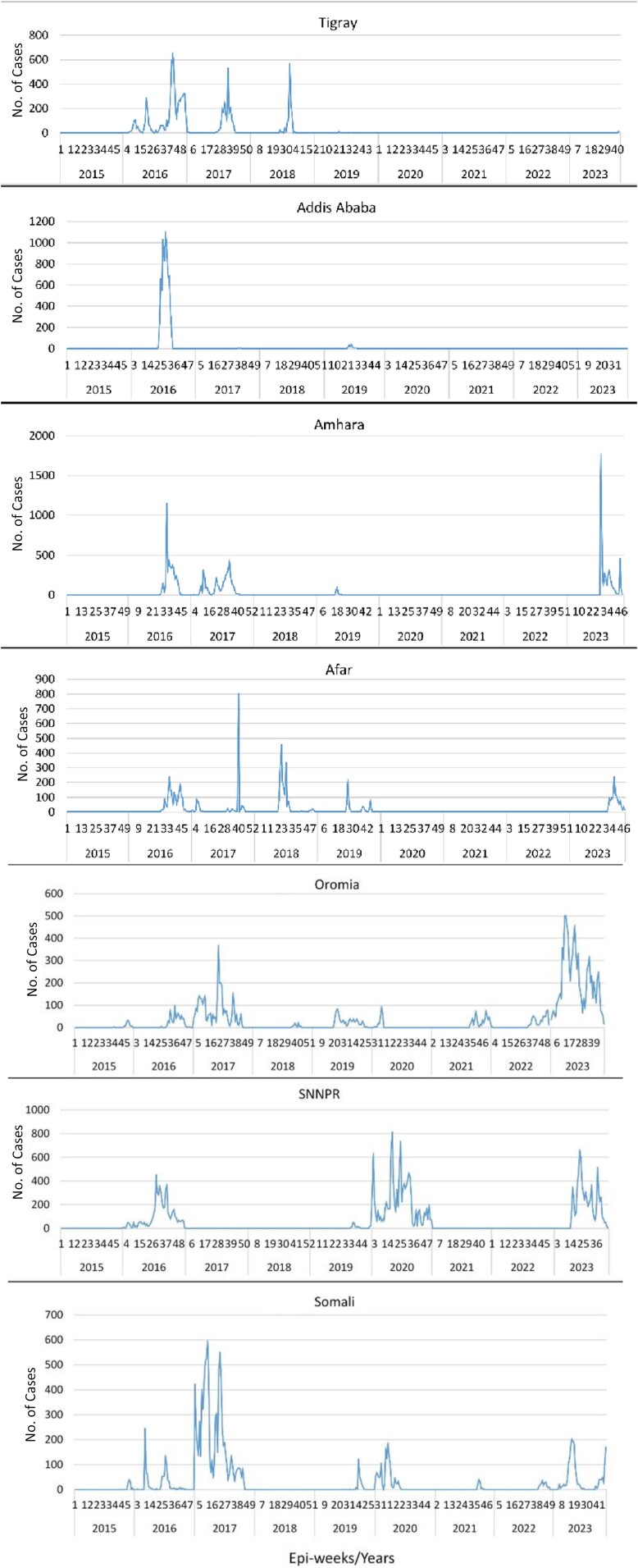
Cholera epidemic curves per region and years in Ethiopia. Graphs show the distributions of cholera cases per year and epi-weeks in each region of Ethiopia during 2015–2023. Abbreviation: SNNPR, Southern Nation, Nationalities, and Peoples’ Region.

The highest number of cholera cases was reported from the Oromia (22 688/99 945 [22.7%]), Southern Nation, Nationalities, and Peoples’ (SNNPR; 20 119/99 945 [20.1%]), Somali (19 010/99 945 [19.0%]), and Amhara (13 366/99 945 [13.4%]) regions, followed by Addis Ababa (7903/99 945 [7.9%]) (Table 2). Regional distribution of cholera cases had heterogeneous temporal and spatial features (Figure 2). The northern and central regions (Amhara, Afar, Tigray, and Addis Ababa) reported cases only till 2019 and were endemic, except in Addis Ababa. However, in the southern and eastern parts (Oromia and Somali regions), it was endemic throughout the study periods. In 2023, the cholera outbreak involved all regions, except Addis Ababa and Gambela. Cholera cases were persistently reported throughout the year in some regions such as Oromia, SNNPR, and Somali in the 2017, 2020, and 2023 outbreaks. The highest number of cases were reported in 2016, 2017, and 2023 with >1000 cases weekly, and the lowest number of cases were reported during 2021 and 2022 with <100 cases per week (Figure 2). During the outbreak years, cholera peaked in the major rainy season of June–August (Kiremt) and reached the lowest during the dry season of December–February (Bega). The 2021 and 2022 outbreaks also occurred following the rainy season after cholera-free periods (Figure 1*B*). The trend showed its endemic nature with cases reported in each year with different magnitude and persistence.

Of the reported 99 945 cholera/AWD cases, 84 206 (84.3%) had dehydration, which included 47 866 (47.9%) with severe dehydration and 36 340 (36.4%) with some dehydration (Table 2). These cholera/AWD patients were predominantly clinically diagnosed (86 080/99 945 [86.1%]) and the rest were confirmed cholera positive by rapid diagnostic test (RDT; 7118/99 945 [7.1%]), culture (851/99 945 [0.90%]), and both RDT and culture (52/99 945 [0.1%]) (Table 2). Eight percent (8005/99 945) of the patients had their stool samples collected for cholera diagnosis (Table 2). Of the samples taken, 91.3% were cholera RDT positive and 73.3% were stool culture positive (Table 2).

### Trends of Cholera Incidence in Ethiopia Categorized by Region, Sex, and Age

**Figure 3.**
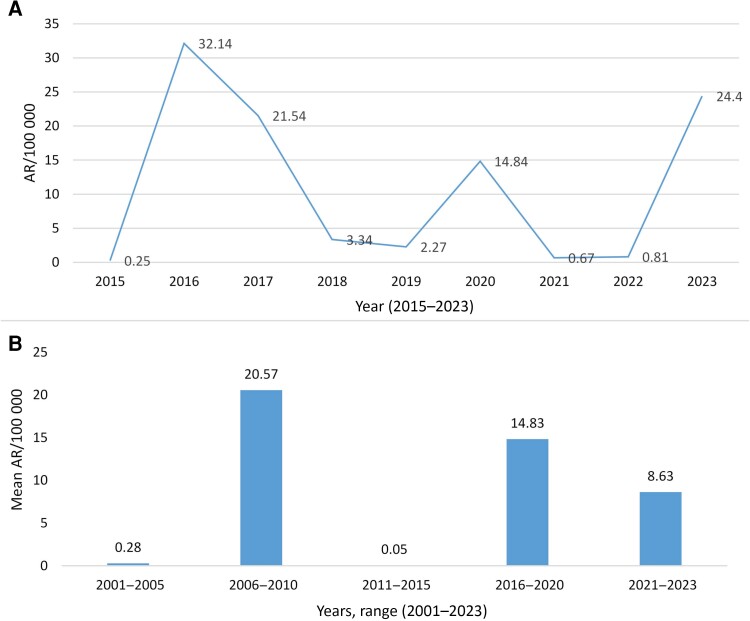
Annual and mean cholera incidence in Ethiopia. *A*, Annual trends of cholera incidence per 100 000 population in Ethiopia during 2015–2023. *B*, Trend of mean cholera incidence per 5-year period during 2001–2023 in Ethiopia. Abbreviation: AR, attack rate.

The cholera incidence rate varied in each reported year with a decreased trend in the last 2 decades until 2022. The incidence peaked in 2023 to among the highest recorded level. Nationally, the highest cholera incidence rates were reported in 2006 (48.24 per 100 000) with subsequent peaks in 2009 (28.11/100 000), 2016 (32.14/100 000), 2017 (21.54/100 000), 2020 (14.84/100 000), and 2023 (24.4/100 000) (Table 1 and Figure 3*A*). The mean incidence rates of cholera/AWD from 2001 until 2023 showed the years 2006–2010 and 2016–2020 with distinct epidemics: 20.57 and 14.83 per 100 000, respectively (Figure 3*B*). Figure 3*B* also shows the recent decrease in mean incidence rate (8.63 per 100 000 during 2021-2023) after OCV introduction in 2019.

Region-stratified incidence rates (per 100 000) from 2015 until 2023 showed the large-scale cholera/AWD outbreaks in Addis Ababa (230.42), Harari (114.71), Somali (106.68), and Tigray (64.61) in 2016; Somali (160.04), Afar (69.44), Dire Dawa (45.49), and Tigray (39.69) in 2017; Afar (994.83 and 28.76) in 2018 and 2019; SNNPR (62.61), Gambela (44.14), Sidama (22.06), and Somali (21.26) in 2020; and Dire Dawa (103.63), Afar (56.02), Somali (56.02), SNNPR (34.67), and Oromia (23.75) in 2023 (Table 3). Overall, cholera/AWD outbreaks hit Tigray region in 2016 and phased out during 2017–2019; started and persisted in Afar region in 2016–2017, peaked in 2018, and phased out in 2019; peaked in Addis Ababa in 2016 and another outbreak in 2019; started in Somali region in 2015, peaked in 2016–2017, and reoccurred in 2019–2023; peaked in Harari region in 2016 and reoccurred in 2019 and 2023; and hit Gambela region in 2020 (Table 3). In Oromia region, cholera/AWD incidences persisted every year throughout 2015–2023 (Table 3).

**Table 3.** Trends of Annual Cholera Incidence Rate per 100 000 and Case Fatality Rate Categorized by Sex, Age, and Region in Ethiopia During 2015–2023

Variables	Incidence Rate/100 000 Population									CFR (%)									Total
	2015	2016	2017	2018	2019	2020	2021	2022	2023	2015	2016	2017	2018	2019	2020	2021	2022	2023	
Sex																			
Male	0.26	38.69	23.67	3.99	2.66	15.98	0.72	0.82	27.10	1.74	0.64	0.82	1.22	1.1	1.6	1.11	3.59	1.71	1.14
Female	0.26	27.32	20.75	2.93	2.07	15.01	0.68	0.88	22.45	0	0.6	0.63	1.38	1.2	1.3	1.19	2.70	0.99	0.9
Age, y																			
0–4	0.53	17.20	17.56	3.35	1.84	16.63	0.84	1.39	24.95	0	1.03	1.06	2.72	2.0	1.3	0.85	4.74	1.39	1.33
5–14	0.11	17.41	16.97	2.25	1.75	142.52	0.81	0.94	20.03	0	0.59	0.69	1.32	1.4	1.3	0.99	2.92	1.13	0.90
15–44	0.23	41.91	24.29	4.16	2.75	17.17	0.61	0.65	27.52	2.1	0.46	0.49	0.65	0.8	1.2	1.39	0.6	0.95	0.73
≥45	0.33	51.10	31.47	3.64	2.90	11.33	0.68	0.66	25.20	0	0.97	1.24	2.47	4.1	3.4	1.14	8.91	3.01	1.84
Region																			
Addis Ababa	0.00	230.42	0.58	0.00	4.36	0.00	0.00	0.00	0.00	0	0.17	0	n.a	0	n.a	n.a	n.a	n.a	0.16
Afar	0.00	77.38	69.44	994.83	28.76	0.00	0.00	0.00	56.02	0	0.8	0.40	0.18	0.5	n.a	n.a	n.a	1.12	1.04
Amhara	0.00	21.37	19.42	0.00	0.87	0.00	0.00	0.00	19.95	0	0	1.53	n.a	2.6	n.a	n.a	n.a	1.68	1.09
B/Gumz	0.00	38.07	6.67	0.00	0.00	0.00	0.00	0.00	14.16	0	5.58	2.82	n.a	n.a	n.a	n.a	n.a	8.47	6.07
Dire Dawa	0.00	11.04	45.49	7.31	1.22	0.00	0.00	0.00	103.63	0	0	0.94	0	0	n.a	n.a	n.a	0.70	0.69
Gambela	0.00	0.00	0.00	0.00	0.00	44.14	0.00	0.00	0.00	0	0	0	n.a	n.a	1.9	n.a	n.a	n.a	1.89
Harari	0.00	114.71	0.00	0.00	5.84	0.00	0.00	0.00	24.38	0	0	0	n.a	0	n.a	n.a	n.a	1.43	0.28
Oromia	0.36	15.56	10.06	0.22	2.30	4.59	1.51	1.66	23.75	0.81	1.12	0.64	0	1.2	1.2	0.85	2.26	1.23	1.10
Sidama	n.a^a^	n.a^a^	n.a^a^	n.a^a^	n.a^a^	22.06	0.00	0.00	24.42	0	0	0	n.a	2.2	2.6	n.a	n.a	0.93	1.42
SNNPR^b^	0.00	5.06	0.00	0.00	0.36	62.61	0.00	0.00	34.67	0	1.06	n.a	n.a	2.7	1.4	n.a	n.a	1.21	1.34
Somali	2.02	106.68	160.04	0.00	4.25	21.26	1.68	3.06	56.02	0.91	0.47	0.43	n.a	1.2	1.4	2.8	6.03	1.84	0.75
Tigray	0.00	64.61	39.69	23.78	0.57	0.00	0.00	0.00	0.411	0	1.26	0.72	0.71	0	n.a	n.a	n.a	n.a	1.00

Abbreviations: y, years; B/Gumz, Benishangul Gumuz; CFR case fatality rate; n.a, not applicable; SNNPR, Southern Nation, Nationalities, and Peoples’ Region.

^a^The administrative region organized later in 2020.

^b^In 2023, administratively divided into 3 regions.

### Trend of Cholera CFR in Ethiopia Categorized by Sex, Age, and Region

**Figure 4.**
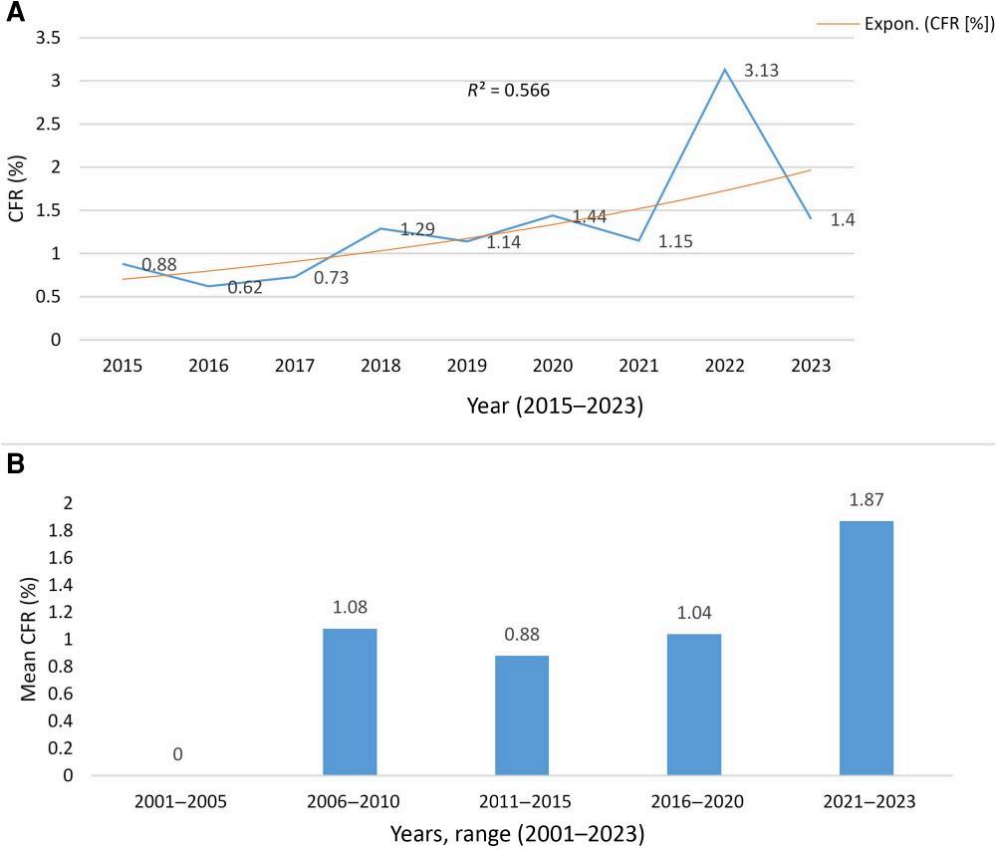
Annual and mean cholera case fatality rate in Ethiopia. *A*, Annual trends of cholera case fatality rate (CFR) in Ethiopia during 2015–2023. *R*^2^ is the coefficient of determination and the best trendline determined using Expon. (CFR [%], exponential trendline). *B*, Trend of mean cholera CFR per 5-year period during 2001–2023 in Ethiopia.

Since 2015, 99 945 cases and 1030 deaths with a cumulative CFR of 1.03% (95% CI: 1.02–1.04) were documented (Table 1). The highest CFR (3.13% [95% CI: 2.1–4.5]) was reported in 2022 (Table 1 and Figure 4*A*). In general, cholera CFR trend showed an increased pattern (Figure 4*A*). The CFRs sharply increased in 2021–2023 (1.87%), especially in 2022 (>3%), which could be attributable to the high cholera CFR in Somali region (6.03%) and Oromia region (2.26%) in 2022 (Table 3).

Regional cholera CFR varied per year during 2015–2023. Overall, the Benishangul Gumuz (B/Gumz) region exhibited the highest cholera CFR (6.07%), followed by Gambela (1.89%), Sidama (1.42%), SNNPR (1.34%), Oromia (1.10%), Amhara (1.09%), Afar (1.04%), Tigray (1.00%), Somali (0.75%), Dire Dawa (0.69%), Harari (0.28%), and Addis Ababa (0.16%) (Table 3 and Table 4). The cholera CFR in 2015 was reported only in Oromia and Somali regions; in 2016 driven largely by B/Gumz (5.58%), Tigray (1.26%), Oromia (1.12%), and SNNPR (1.06%) regions; in 2017 by B/Gumz (2.82%) and Amhara (1.53%) regions; in 2019 by SNNPR (2.7%), Amhara (2.6%), Sidama (2.2%), Oromia (1.2%), and Somali (1.2%) regions; in 2020 by Sidama (2.6%), Gambela (1.9%), SNNPR (1.4%), Somali (1.4%), and Oromia (1.2%); in 2021 by Somali (2.8%) and Oromia (0.85%); in 2022 by Somali (6.03%) and Oromia (2.26%); and in 2023 by Somali (1.84%), Oromia (1.43%), and SNNPR (1.21%). In the recent 2023 outbreak, the highest cholera CFR was recorded in B/Gumz (8.47%) (Table 3). Among the districts or zones of a region, the cholera CFR also differed (Supplementary Table 1).

**Table 4.** Annual Cholera Cases, Deaths, and Case Fatality Rate Categorized by Sex, Age, and Region in Ethiopia During 2015–2023

Category	Cases, No.										Deaths (CFR), No. (%)									
	2015	2016	2017	2018	2019	2020	2021	2022	2023	Total	2015	2016	2017	2018	2019	2020	2021	2022	2023	Total
Sex																				
Male	115	17 572	10 969	1884	1281	7844	360	418	14 861	55 299	2 (1.74)	112 (0.64)	90 (0.82)	23 (1.22)	14 (1.1)	124 (1.6)	4 (1.11)	15 (3.59)	249 (1.71)	633 (1.14)
Female	115	12 299	9540	1375	990	7323	336	444	12 240	44 646	0 (0)	74 (0.6)	60 (0.63)	19 (1.38)	12 (1.2)	95 (1.3)	4 (1.19)	12 (2.70)	121 (0.99)	397 (0.90)
Age, y																				
0–4	71	2333	2269	441	247	2278	117	190	3517	11 460	0 (0)	24 (1.03)	24 (1.06)	12 (2.72)	5 (2.0)	29 (1.3)	1 (0.85)	9 (4.74)	49 (1.39)	153 (1.33)
5–14	25	4095	3916	529	420	3553	203	240	5 293	18 273	0 (0)	24 (0.59)	27 (0.69)	7 (1.32)	6 (1.4)	46 (1.3)	2 (0.99)	7 (2.92)	60 (1.13)	179 (0.90)
15–44	96	17 435	10 540	1843	1242	7895	288	331	14.336	53 990	2 (2.1)	80 (0.46)	52 (0.49)	12 (0.65)	10 (0.8)	95 (1.2)	4 (1.39)	2 (0.6)	142 (0.95)	399 (0.73)
≥45	38	6008	3777	446	362	1441	88	101	3955	16 215	0 (0)	58 (0.97)	47 (1.24)	11 (2.47)	15 (4.1)	49 (3.4)	1 (1.1.4)	9 (8.91)	119 (3.01)	299 (1.84)
Region																				
Addis Ababa	0	7726	20	0	157	0	0	0	0	7903	0 (0)	13 (0.17)	0 (0)	n.a	0 (0)	n.a	n.a	n.a	n.a	13 (0.16)
Afar	0	1368	1259	1874	547	0	0	0	1163	6206	0 (0)	11 (0.8)	5 (0.40)	33 (0.18)	3 (0.5)	n.a	n.a	n.a	13 (1.12)	65 (1.04)
Amhara	0	4438	4105	0	191	0	0	0	4632	13 366	0 (0)	0 (0)	63 (1.53)	n.a	5 (2.6)	n.a	n.a	n.a	78 (1.68)	146 (1.09)
B/Gumz	0	394	71	0	0	0	0	0	177	642	0 (0)	22 (5.58)	2 (2.82)	n.a	n.a	n.a	n.a	n.a	15 (8.47)	39 (6.07)
Diredawa	0	50	212	35	6	0	0	0	570	873	0 (0)	0 (0)	2 (0.94)	0 (0)	0 (0)	n.a	n.a	n.a	4 (0.70)	6 (0.69)
Gambela	0	0	0	0	0	211	0	0	0	211	0 (0)	n.a	n.a	n.a	n.a	4 (1.9)	n.a	n.a	n.a	4 (1.89)
Harari	0	273	0	0	15	0	0	0	69	357	0 (0)	0 (0)	n.a	n.a	0 (0)	n.a	n.a	n.a	1 (1.40)	1 (0.28)
Oromia	120	5379	3569	79	857	1752	589	663	9710	22 688	1 (0.81)	60 (1.12)	23 (0.64)	0 (0)	10 (1.2)	21 (1.2)	5 (0.85)	15 (2.26)	119 (1.43)	251 (1.10)
Sidama	0	0	0	0	134	535	0	0	1295	1833	0 (0)	NA	n.a	n.a	3 (2.2)	14 (2.6)	n.a	n.a	12 (0.93)	26 (1.42)
SNNPR	0	947	0	0	73	11 350	0	0	7615	20 119	0 (0)	10 (1.06)	n.a	n.a	2 (2.7)	162 (1.4)	n.a	n.a	92 (1.21)	269 (1.34)
Somali	110	5973	9199	0	257	1319	107	199	1846	19 010	1 (0.91)	28 (0.47)	40 (0.43)	n.a	3 (1.2)	18 (1.4)	3 (2.8)	12 (6.03)	34 (1.84)	142 (0.75)
Tigray	0	3328	2083	1271	31	0	0	0	24	6737	0 (0)	42 (1.26)	15 (0.72)	9 (0.71)	0 (0)	n.a	n.a	n.a	2 (8.3)	68 (1.00)

Abbreviations: y, years; B/Gumz, Benishangul Gumuz; CFR case fatality rate; n.a, not applicable; SNNPR, Southern Nation, Nationalities, and Peoples’ Region.

During 2015–2023, the cholera CFR was slightly higher in men (1.14%) than women (0.90%) (Table 3 and Table 4). The age-stratified cholera CFRs exhibited the older adults (aged ≥45 years) and young children (<5 years) at higher risk with CFR of 1.84% and 1.33%, respectively (Table 3). Notably, the cholera CFR of 8.91% in older adults (aged ≥45 years), 4.74% in young children (<5 years), and 2.92% in older children (aged between 5 and 14 years) were exhibited all in 2022. The cholera CFRs in all age groups were persistent at similar levels almost every year with particular increase of CFRs in older adults (aged ≥45 years) in 2019 (4.1%) and 2022 (8.91%); in young children (<5 years) in 2018 (2.72%), 2019 (2.0%), and 2022 (4.74%); in older children (5–14 years) in 2019 (1.4%) and 2022 (2.92%); and in adolescents and young adults (15–44 years) in 2015 (2.1%), 2020 (1.2%), and 2021 (1.39%) (Tables 3 and 4).

### Determinants of Cholera CFR

During the multivariate logistic regression analysis to identify the CFR risks, we found that after adjusting for age, treatment modality, dehydration level, and outbreak season, the odds of death from cholera decreased by 20% for female compared to male (adjusted odds ratio [AOR], 0.79 [95% CI: .69–.89]) reported cases (Table 5). Cholera/AWD patients in older adults aged ≥45 years (AOR, 1) were at higher risk of deaths compared to the younger age groups. Those aged 5–14 years (AOR, 0.55 [95% CI: .45–.66]) and 15–44 years (AOR, 0.41 [95% CI: .35–.48]) were nearly 50% less likely to die due to cholera compared to older age groups. Similarly, patients with clinical manifestation of no or some dehydration were nearly 70% less likely to die than those with severe dehydration (AOR, 1): no dehydration (AOR, 0.13 [95% CI: .09–.18]), and some dehydration (AOR, 0.23 [95% CI: .19–.28]). In terms of seasonality, we found that the highest cholera CFR was reported during the peak outbreak seasons. Compared to the December–February season with lower cholera cases, March–May (AOR, 1.89 [95% CI: 1.43–2.49]), June–August (AOR, 1.63 [95% CI: 1.25–2.13]), and September–November (AOR, 1.50 [95% CI: 1.14–1.99]) reported over 50% risk of death related to cholera. Patients treated at the outpatient level had higher risk of mortality (AOR, 1.40 [95% CI: 1.19–1.64]) than those admitted to a cholera treatment center. We investigated the possible CFR risk difference of recent years after observing the recent high CFR. During 2019–2023, the magnitude and identified risk factors associated with cholera CFR were similar to the above findings, except in the treatment modality. In the recent years, the risk of death in cholera outpatient treatment modality was >2 times (AOR, 2.69 [95% CI: 1.99–3.62]) (Table 5).

**Table 5.** Determinants of Cholera Case Fatality Rate in Ethiopia

Variable	Category	2015–2023					2019–2023				
		Outcome		CFR, %	COR (95% CI)	AOR^a^ (95% CI)	Outcome		CFR, %	COR (95% CI)	AOR (95% CI)
		Alive, No.	Death, No.				Alive, No.	Death, No.			
Sex	Female	44 249	397	0.90	0.78 (.68–.88)	0.79 (.69–.89)	21 073	244	1.10	0.69 (.59–.82)	0.72 (.61–.85)
	Male	54 666	633	1.14	1	1	24 353	406	1.59	1	1
Age	<5 y	11 307	153	1.33	0.72 (.59–.88)	0.84 (.68–1.02)	6255	93	1.56	0.47 (.36–.60)	0.59 (.46–.76)
	5–14 y	18 094	179	0.94	0.53 (.44–.64)	0.55 (.45–.66)	9587	121	1.35	0.40 (.32–.50)	0.45 (.35–.57)
	15–44 y	53 591	399	0.73	0.40 (.34–.46)	0.41 (.35–.48)	23 823	253	1.10	0.33 (.28–.41)	0.37 (.29–.45)
	≥45 y	15 916	299	1.84	1	1	5761	183	3.09	1	1
Dehydration status	Unknown	2043	34	1.66	0.96 (.68–1.36)	1.12 (.79–1.59)	1976	34	1.72	0.70 (.40–.99)	0.83 (.56–1.19)
	No	13 629	34	0.29	0.14 (.10–.20)	0.13 (.09–.18)	4616	21	0.41	0.18 (.11–.27)	0.11 (.07–.17)
	Some	36 191	149	0.44	0.24 (.20–.28)	0.23 (.19–.28)	18 663	97	0.5	0.21 (.17–.26)	0.17 (.14–.22)
	Severe	47 052	814	1.71	1	1	20 171	499	2.41	1	1
Treatment modality	Outpatient	20 449	199	1.0	0.93 (.80–1.09)	1.40 (1.19–1.64)	5458	67	1.21	0.17 (.91–1.52)	2.69 (1.99–3.62)
	Inpatient	77 022	804	1.0	1	1	38 708	558	1.42	1	1
Outbreak season	March–May (Belg)^b^	20 765	250	1.20	1.83 (1.39–2.40)	1.89 (1.43–2.49)	12 874	168	1.31	1.12 (.81–1.54)	1.11 (.81–1.55)
	Jun–Aug (Kirmt)^c^	42 801	435	1.0	1.55 (1.19–2.00)	1.63 (1.25–2.13)	17 258	248	1.4	1.231 (.91–1.68)	1.24 (.90–1.71)
	Sept–Nov (Mehir)^d^	25 315	279	1.1	1.68 (1.28–2.19)	1.50 (1.14–1.99)	11 096	185	1.6	1.43 (1.04–1.96)	1.32 (.95–1.84)
	Dec–Feb (Bega)^e^	10 034	66	0.70	1	1	4198	49	1.20	1	1

Abbreviations: COR, crude odds ratio; AOR, adjusted odds ratio; CFR, case fatality rate; CI, confidence interval; y, years.

^a^Multivariate logistic regression outcomes of sex, age, dehydration status, treatment modality, and outbreak season.

^b^Spring with a relatively rainy season in some parts of the country.

^c^Summer with a major rainy season all over the country.

^d^Spring with a relatively dry season.

^e^Autumn with a dry season.

## DISCUSSION

The cholera outbreak patterns in Ethiopia showed annual cases persistently during the past two decades, except for 4–5 cholera-free years over ten years ago. Similar outbreak patterns were reported in neighboring South Sudan, where three epidemic waves were reported during 2014–2017 with periods of no cases between waves [17]. This alludes to possible cross-border transmission and the importance of controlling cholera in nearby countries through a coordinated intervention strategy. Since 2015, cholera outbreaks have been reported every year with different magnitude. Cholera peaks during 2016–2017 in Ethiopia were associated with the 2015–2016 El Niño effect, which also affected other countries in Africa especially the East African region [18]. In line with the recent global cholera resurgence after several years of decline [19], cholera cases increased again in Ethiopia in 2023 particularly during the major rainy season of June–August, lasting around 29–39 epi-weeks annually. The cholera cases in 2021–2022 were reported after the major rainy seasons, which may be associated with fecal contamination of drinking water source due to flooding. Strengthening cholera prevention interventions before the rainy season is critical. Larger cholera attack rates were considered to be associated with longer epidemic durations [20]. We found similar patterns whereby persistent cholera outbreaks were shown throughout the epi-year when over 200 and 500 cases per week at regional- and national-levels respectively were reported. This could be a proxy indicator for a prolonged outbreak, and appropriate resource allocations and cholera control interventions should be thought through.

We observed two distinct upsurges in the mean incidence of cholera/AWD during the years 2006–2010 and 2016–2020, and recent epidemics were reported in 2019–2023. In response to the recent outbreaks, the Ethiopian government has conducted several reactive mass OCV vaccination campaigns since 2019. As part of the global cholera elimination target by 2030, the Ethiopian NCP was developed and has been implemented since 2022 [11]. Further research is warranted to evaluate the impact, effectiveness, and efficiency of the various interventions implemented in order to better understand their associations with cholera outbreak controls and preventions. During the COVID-19 pandemic and the cholera outbreaks in 2020–2021, the COVID-19 preventive interventions such as the promotion of hand washing and hygiene, physical distancing, and banning of gatherings could have contributed to the relatively lower number of cholera cases in these years. However, this needs to be investigated properly as there could have been also many unreported or underreported cases of cholera [21], especially if local populations had modified cholera-associated healthcare-seeking behavior during the COVID-19 pandemic per different regions of the country.

The cholera CFR is an indicator of the quality of cholera case management, and the recommended level is <1% [22]. We found a cumulative CFR of 1.09% for the past 2 decades (2001–2023), 1.03% for 2015–2023, and an increased cumulative CFR of 1.87% for 2021–2023 in Ethiopia. Global cholera mortality has been increasing in Africa; from 8.7/100 000 in 1990 to 11.3/100 000 in 2019 with a 1.2% annual increment [23]. The cholera CFR in Ethiopia has not yet reached the NCP target, which aimed a 30% decrease by 2023 compared to the baseline of 1.8% [11, 24]. The highest cholera CFR (3.13%) in Ethiopia occurred during the 2022 outbreak and the CFR varied per regions. Cholera CFR tend to be higher at the early phase of outbreak according to a study in Haiti [25] and different across districts as shown in Uganda [26].

We further found that people age ≥45 years, followed by age <5 years, severe dehydration, outpatient level management, and rainy season outbreaks were the high risk factors associated with cholera CFR in Ethiopia. A study in Uganda also showed that older age groups had a higher mortality rate than younger age groups [26]. It is well known that the cause of death related to cholera is severe dehydration [27]. Our finding has programmatic advantages in terms of resource allocation. Prioritizing older patients in vaccination programs could be one strategic approach, especially in areas with high cholera CFR in older age groups, if OCV doses are in shortage. Preparation for future cholera outbreaks should be planned sufficiently before the major rainy season in Ethiopia each year to effectively reduce the cholera related deaths. Community awareness and engagement for active case finding and referral to cholera treatment center/unit or health centers are important to contain outbreaks and for timely case management [28]. In our Ethiopia Cholera Control and Prevention (ECCP) project, local populations in Shashemene area had relatively good knowledge and disease perception towards cholera [29]. Areas such as this, where cholera has been endemic and persistently reported may contribute to the high level of community awareness on cholera. The nonhospitalized cholera case management needs to be revisited to reduce the cholera deaths among outpatients. Appropriate cholera diagnostic capacity should be guaranteed at health centers or cholera treatment centers/units close to the affected areas, and follow-up of outpatients at the community level needs to be considered to reduce the CFR in this patient group. Sustainable resources are required to strengthen cholera surveillance system, including early case detection, health information management and reporting, laboratory confirmation and prepositioning of supplies such as RDT kits, laboratory supplies, and oral rehydration solution (ORS).

This study has some limitations. First, we used the WHO incidence and CFR gross data before 2015. This limited us in assessing the detailed epidemiological characteristics of the outbreaks in those earlier years. Second, the reported cases might not reflect the true cholera incidence in Ethiopia due to underreporting. Third, seasonality was analyzed based on typical four major seasons in Ethiopia, but historical climate records on droughts and floodings were not considered in our analysis. Fourth, we only presented the cholera CFRs at regional- and national-levels without any investigations on the causes of CFR variations between regions. Further research is recommended on the region/district/site-specific cholera case management practice and outbreak control and preventive interventions to identify the critical gaps and pathways toward reducing cholera-related deaths.

## CONCLUSIONS

Cholera is an important public health concern in Ethiopia. In the past two decades, two major upsurges of cholera epidemics (2006–2010 and 2016–2020) and another resurgence of outbreaks in 2021–2023 were noted, with the highest cholera CFR. Cholera case management needs to be improved particularly in outpatients and older populations. Cholera outbreak preparedness programs should be rolled out well in advance of the typical rainy seasons. Significant investments are essential to advance the cholera surveillance system at healthcare setting and community level. Underlying factors of cholera deaths per areas should be further investigated to guide appropriate interventions to meet the NCP target by 2028.

## Supplementary Data

Supplementary material is available at *Clinical Infectious Diseases* online. Consisting of data provided by the authors to benefit the reader, the posted materials are not copyedited and are the sole responsibility of the authors, so questions or comments should be addressed to the corresponding author.

## Supplementary Material

ciae236_Supplementary_Data

## Notes

***Author contributions.*** A. G., Y. W. D., M. T., and S. E. P. conceptualized the overall manuscript. Database review, data analysis, and manuscript drafting were led by A. G. with Y. W. D. in discussion with M. T. and S. E. P. All authors read and provided feedback and approved the final draft.

***Acknowledgments.*** The authors acknowledge the Ethiopian Public Health Institute for the access to national health data system.

***Data sharing.*** All data relevant to the study are included in this manuscript.

***Financial support*.** This study was supported by the Korea Support Committee for International Vaccine Institute (CHMTD05083-010), LG Electronics (CHMTD05083-020), and Community Chest of Korea (CHMTD05083-030). The International Vaccine Institute (IVI) acknowledges its donors, including the government of the Republic of Korea and the Swedish International Development Cooperation Agency.

***Supplement sponsorship.*** This article appears as part of the supplement “Ethiopia Cholera Control and Prevention (ECCP): Evidence-Generation Towards Global Roadmap to Ending Cholera,” sponsored by the IVI.

## References

[1] Ali M , Nelson AR, Lopez AL, Sack DA. Updated global burden of cholera in endemic countries. PLoS Negl Trop Dis 2015; 9:e0003832. 26043000 10.1371/journal.pntd.0003832 PMC4455997

[2] Ali M , Lopez AL, You YA, et al The global burden of cholera. Bull World Health Organ 2012; 90:209–18. 22461716 10.2471/BLT.11.093427 PMC3314202

[3] Ganesan D , Gupta SS, Legros D. Cholera surveillance and estimation of burden of cholera. Vaccine 2020; 38:A13–7. 31326254 10.1016/j.vaccine.2019.07.036

[4] Pankhurst R. The history of cholera in Ethiopia. Med Hist. 1968; 12:262–9. 10.1017/s0025727300013302 PMC1033828 4875613

[5] Scrascia M, Pugliese N, Maimone F, Mohamud KA, Ali IA, Grimont PA, Pazzani C. Cholera in Ethiopia in the 1990s: epidemiologic patterns, clonal analysis, and antimicrobial resistance. Int J Med Microbiol 2009; 299:367–72. 10.1016/j.ijmm.2008.10.004 19121605

[6] Moore S, Worku Demlie Y, Muluneh D, et al. Spatiotemporal dynamics of cholera epidemics in Ethiopia: 2015-2021. Sci Rep 2024; 14:7170. 10.1038/s41598-024-51324-z PMC10991303 38570534

[7] Park SE, Jeon Y, Kang S, et al. Infectious disease control and management in Ethiopia: A case study of Cholera. Front Public Health 2022; 10:870276. 10.3389/fpubh.2022.870276 PMC9197421 35712321

[8] Ethiopia Health and Nutrition Research Institute . Guideline on cholera outbreak management Ethiopia. Addis Ababa: Ethiopia Health and Nutrition Research Institute, 2011.

[9] Ethiopian Public Health Institute . National guideline for cholera surveillance and outbreak response. 3rd ed. Addis Ababa: Ministry of Health and Ethiopian Public Health Institute, 2022.

[10] Edosa M, Jeon Y, Gedefaw A, et al . Comprehensive review on the use of oral Cholera vaccine (OCV) in Ethiopia: 2019 to 2023. Clin Infect Dis 2024; 79 (S1):S20–32. 38996038 10.1093/cid/ciae200 PMC11244260

[11] Ethiopian Public Health Institute . Multi-sectorial cholera elimination plan: Ethiopia 2021–2028. Addis Ababa: Ethiopian Public Health Institute, 2021.

[12] The World Bank database . Available at: https://data.worldbank.org/indicator/EN.POP.DNST?locations=ETIL. Accessed 24 May 2024.

[13] Girma M , Hussein A, Norris T, et al Progress in water, sanitation and hygiene (WASH) coverage and potential contribution to the decline in diarrhea and stunting in Ethiopia [manuscript published online ahead of print 4 November 2021]. Maternal Child Nutr 2021. doi:10.1111/mcn.13280 PMC11258769 34738323

[14] Jerving S . Why governments tiptoe around the word ‘cholera.’ 2018. Available at: https://www.devex.com/news/why-governments-tiptoe-around-the-word-cholera-92348. Accessed 23 November 2023.

[15] World Health Organization . Cholera cases 2000–2021: WHO-dataset. Geneva, Switzerland: WHO, 2022.

[16] Central Statistical Agency . Population projections for Ethiopia 2007–2037. Addis Ababa, Ethiopia: Central Statistical Agency, 2013.

[17] Jones FK , Wamala JF, Rumunu J, et al Successive epidemic waves of cholera in South Sudan between 2014 and 2017: a descriptive epidemiological study. Lancet Planet Health 2020; 4:e577–87. 33278375 10.1016/S2542-5196(20)30255-2 PMC7750463

[18] Moore SM , Azman AS, Zaitchik BF, et al El Niño and the shifting geography of cholera in Africa. Proc Natl Acad Sci U S A 2017; 114:4436–41. 28396423 10.1073/pnas.1617218114 PMC5410791

[19] Larkin H . WHO report: cholera resurgent in 2022 after years of decline. JAMA 2023; 329:200. 10.1001/jama.2022.23551 36648460

[20] Zheng Q , Luquero FJ, Ciglenecki I, et al Cholera outbreaks in sub-Saharan Africa during 2010–2019: a descriptive analysis. Int J Infect Dis 2022; 122:215–21. 35605949 10.1016/j.ijid.2022.05.039 PMC9439956

[21] Uwishema O , Okereke M, Onyeaka H, et al Threats and outbreaks of cholera in Africa amidst COVID-19 pandemic: a double burden on Africa's health systems. Trop Med Health 2021; 49:93. 34819175 10.1186/s41182-021-00376-2 PMC8611385

[22] World Health Organization . Cholera. Update, end of 1993. Wkly Epidemiological Rec 1994; 69:13–7.

[23] Ilic I , Ilic M. Global patterns of trends in cholera mortality. Trop Med Infect Dis 2023; 8:169. 36977170 10.3390/tropicalmed8030169 PMC10058923

[24] Hussen M, Worku Demlie Y, Edosa M, et al. Ethiopia National Cholera Elimination Plan 2022–2028: experiences, challenges, and the way forward. Clin Infect Dis **2024**; 79(S1):S1–7. 10.1093/cid/ciae200 PMC11244260 38996038

[25] Barzilay EJ , Schaad N, Magloire R, et al Cholera surveillance during the Haiti epidemic—the first 2 years. N Engl J Med 2013; 368:599–609. 23301694 10.1056/NEJMoa1204927

[26] Bwire G , Malimbo M, Maskery B, Kim YE, Mogasale V, Levin A. The burden of cholera in Uganda. PLoS Negl Trop Dis 2013; 7:e2545. 24340106 10.1371/journal.pntd.0002545 PMC3855006

[27] Dutta P , Sur D, Bhattacharya S. Management of cholera. Epidemiological Mol Aspects Cholera 2011; 1:341–53.

[28] Ohene S-A , Klenyuie W, Sarpeh M. Assessment of the response to cholera outbreaks in two districts in Ghana. Infect Dis Poverty 2016; 5:71–81. 27802834 10.1186/s40249-016-0192-z PMC5090876

[29] Getahun T, Hailu D, Mogeni OD, et al. Healthcare seeking behavior and disease perception toward cholera and acute diarrhea among populations living in cholera high-priority hotspots in Shashemene, Ethiopia. Clin Infect Dis 2024; 79 (S1):S43–52. 38996036 10.1093/cid/ciae232 PMC11244153

