# Supplementary material for: Retrospective Analysis of Cholera/Acute Watery Diarrhea Outbreaks in Ethiopia From 2001 To 2023: Incidence, Case Fatality Rate, and Seasonal and Multiyear Epidemic Patterns
Source: Clin Infect Dis. 2024 Jul 12;79(Suppl 1):S8–S19. doi: 10.1093/cid/ciae236 (PMC11244192; doi:10.1093/cid/ciae236)
Supplement: ciae236_Supplementary_Data [file ciae236_supplementary_data.docx]

**Supplementary Table 1. Zonal-level CFR distribution difference for the past 5 years in the southern Ethiopia**

|  | **Year** | **2023** | | | **2022** | | | **2021** | | | **2020** | | | **2019** | | |
| --- | --- | --- | --- | --- | --- | --- | --- | --- | --- | --- | --- | --- | --- | --- | --- | --- |
| **Region** | **Zone** | **Case**  **(n)** | **Death**  **(n)** | **CFR**  **(%)** | **Case**  **(n)** | **Death**  **(n)** | **CFR**  **(%)** | **Cases**  **(n)** | **Death**  **(n)** | **CFR**  **(%)** | **Case**  **(n)** | **Deaths**  **(n)** | **CFR**  **(%)** | **Case**  **(n)** | **Deaths**  **(n)** | **CFR**  **(%)** |
| **Oromia** | **Guji** | 2,203 | 30 | 1.4 | 162 | 2 | 1.2 | - | - | - | 252 | 2 | 0.8 | 6 | 0 | 0.0 |
|  | **Bale** | 452 | 4 | 0.9 | 472 | 10 | 2.1 | 551 | 4 | 0.7 | 0 | - | - | 2 | 0 | 0 |
|  | **Borena** | 1,009 | 4 | 0.4 | - | - | - | - | - | - | 138 | 2 | 1.4 | 54 | 1 | 1.9 |
|  | **Burji Special** | 48 | 4 | 8.3 | - | - | - | - | - | - | 0 | - | - | - | - | - |
|  | **West Arsi** | 183 | 0 | 0.0 | 29 | 0 | 0.0 | 38 | 1 | 2.6 | 342 | 3 | 0.9 | 164 | 3 | 1.8 |
|  | **West Guji** | 286 | 5 | 1.7 | - | - | - | - | - | - | 878 | 14 | 1.6 | - | - | - |
|  | **East Bale** | 482 | 15 | 3.1 | - | - | - | - | - | - | 0 | - | - | - | - | - |
|  | **Shashemene Town** | **-** | - | - | - | - | - | - | - | - | 142 | 0 | 0.0 | 56 | 0 | 0.0 |
|  | **East Harerge** | - | - | - | - | - | - | - | - | - | - | - | - | 136 | 3 | 2.2 |
|  | **West Harerge** | - | - | - | - | - | - | - | - | - | - | - | - | 438 | 4 | 0.9 |
| **SNNPR** | **Amaro** | 44 | 2 | 4.5 | - | - | - | - | - | - | - | - | - | - | - | - |
|  | **Gamo** | 535 | 5 | 0.9 | - | - | - | - | - | - | 350 | 4 | 1.1 | - | - | - |
|  | **Gedeo** | 167 | 3 | 1.8 | - | - | - | - | - | - | 1,006 | 7 | 0.7 | - | - | - |
|  | **Gofa** | 136 | 0 | 0.0 | - | - | - | - | - | - | 616 | 6 | 1.0 | 13 | 0 | 0.0 |
|  | **Dawro** | - | - | - | - | - | - | - | - | - | 563 | 11 | 2.0 | - | - | - |
|  | **Alle Sp. Woreda** | 146 | 3 | 2.1 | - | - | - | - | - | - | 112 | 1 | 0.9 | 1 | 0 | 0 |
|  | **Konso** | 13 | 4 | 30.8 | - | - | - | - | - | - | 0 | - | - | - | - | - |
|  | **Bench Sheko** | - | - | - | - | - | - | - | - | - | 237 | 6 | 2.5 | - | - | - |
|  | **South Omo** | 146 | 0 | 0.0 | - | - | - | - | - | - | 3,737 | 47 | 1.3 | 28 | 1 | 3.6 |
|  | **West Omo** | - | - | - | - | - | - | - | - | - | 4,556 | 76 | 1.7 | - | - | - |
| **Sidama** | **Hawassa** | 64 | 0 | 0.0 | - | - | - | - | - | - | 23 | 0 | 0.0 | 96 | 2 | 2.1 |
|  | **Sidama** | - | - | - | - | - | - | - | - | - | 685 | 18 | 2.6 | 38 | 1 | 2.6 |
| **Somalia** | **Afder** | 51 | 0 | 0.0 | - | - | - | - | - | - | 712 | 6 | 0.8 | - | - | - |
|  | **Shebele** | - | - | - | - | - | - | - | - | - | 309 | 9 | 2.9 | - | - | - |
|  | **Liban** | 669 | 10 | 1.5 | 199 | 15 | 7.5 | 107 | 3 | 2.8 | 177 | 2 | 1.1 | - | - | - |
|  | **Agnewak** | - | - | - | - | - | - | - | - | - | 211 | 4 | 1.9 | - | - | - |
|  | **Dawa** | 491 | 5 | 1.0 | - | - | - | - | - | - | 121 | 1 | 0.8 | 213 | 1 | 0.5 |
